# Supplementary material for: Can Psychedelic Use Benefit Meditation Practice? Examining Individual, Psychedelic, and Meditation-Related Factors
Source: medRxiv. 2024 Aug 28:2024.08.27.24312677. Preprint. [Version 1] doi: 10.1101/2024.08.27.24312677 (PMC11383514; doi:10.1101/2024.08.27.24312677)
Supplement: Supplement 1 [file NIHPP2024.08.27.24312677v1-supplement-1.pdf]

Supplemental Table 1

Pearson Correlation Coefficients between the Outcome Variable and each of the Predictors.

Variable	*r*	95 % CI		p_{raw}	p_{FDR}
		LL	*UL*		
Individual Factors					
Age	-.01	-.08	.06	.771	.817
Female	.05	-.02	.11	.164	.217
Education (in years)	-.06	-.13	.01	.077	.123
Life satisfaction	.13	.06	.19	<.001	<.001
Life worthwhile	.13	.07	.20	<.001	<.001
Openness to experience	.16	.10	.23	<.001	<.001
Neuroticism	-.11	-.17	-.04	.002	.006
Conscientiousness	.09	.02	.15	.011	.022
Agreeableness	.15	.09	.22	<.001	<.001
Extraversion	.07	.00	.14	.042	.071
Alcohol use (12 months)	-.01	-.08	.05	.668	.738
Tobacco use (12 months)	.09	.03	.16	.006	.012
Cannabis use (12 months)	.18	-.08	.06	<.001	<.001
Psychedelic Factors					
Psychedelics use (12 months)	.34	.28	.40	<.001	<.001
Psychedelics use (lifetime)	.15	.08	.21	<.001	<.001
Psychedelics starting age	.10	.04	.17	.002	.007
Adequate psychedelics framework	.20	.13	.26	<.001	<.001

Trust in psychedelics context	.19	.12	.25	<.001	<.001
Setting intentions during psychedelic use	.30	.23	.36	<.001	<.001
Safe setting	.22	.16	.29	<.001	<.001
Psilocybin	.06	.00	.13	.064	.107
LSD	.08	.02	.15	.013	.025
Ayahuasca	.14	.07	.20	<.001	<.001
5-MeO-DMT	.17	.11	.24	<.001	<.001
N,N-DMT	.23	.16	.29	<.001	<.001
Mescaline	.09	.03	.16	.005	.012
Other	.10	.04	.17	.003	.008
Meditation Factors					
Years of regular practice	-.01	-.08	.05	.695	.752
Frequency of regular practice	-.05	-.12	.02	.138	.192
Meditation starting age	-.04	-.10	.03	.279	.336
Adequate meditation framework	.08	.01	.14	.023	.044
Retreat experience	-.17	-.23	-.10	<.001	<.001
Retreat days (12 months)	-.10	-.17	-.03	.003	.009
Retreat days (lifetime)	-.05	-.12	.01	.128	.192
Retreat longest	-.07	-.14	-.01	.031	.056
Meditation practice before psychedelic experience	.002	-.06	.07	.944	.962
Burmese Vipassana	-.10	-.16	-.03	.004	.010
Culadasa	-.10	-.16	-.03	.004	.010
Eclectic/other	.11	.04	.18	.001	.004

Goenka Vipassana	-.01	-.07	.06	.826	.858
Hindu practice	.09	.03	.16	.006	.013
Jhana practice	-.04	-.10	.03	.287	.338
Japanese Zen	.04	-.03	.11	.231	.291
Modern mindfulness	.03	-.03	.10	.327	.377
Rob Burbea	-.06	-.13	.01	.080	.125
Sam Harris	.07	.01	.14	.034	.060
Shinzen Young	-.05	-.12	.02	.141	.192
Tibetan Chagzog	.05	-.02	.12	.141	.192
Thich Nhat Hahn	.02	-.05	.08	.620	.700
Tibetan Tantric meditation	-.04	-.11	.02	.199	.257
Western loving kindness compassion	.00	-.07	.07	.980	.980
Western non-dual meditation	.04	-.03	.11	.243	.300
Western Vipassana	-.05	-.12	.02	.137	.192

Note. CI = confidence interval, fdr = False discovery rate; LSD = Lysergic acid diethylamide; 5-MeO-DMT = 5-methoxy-N,N-dimethyltryptamine N,N-DMT = N,N-Dimethyltryptamine, .
